# Tyrosine Y189 in the Substrate Domain of the Adhesion Docking Protein NEDD9 Is Conserved with p130Cas Y253 and Regulates NEDD9-Mediated Migration and Focal Adhesion Dynamics

**DOI:** 10.1371/journal.pone.0069304

**Published:** 2013-07-09

**Authors:** Jaime B. Baquiran, Peta Bradbury, Geraldine M. O'Neill

**Affiliations:** 1 Children’s Cancer Research Unit, Kids Research Institute, The Children’s Hospital at Westmead, Westmead, NSW, Australia; 2 Discipline of Paediatrics and Child Health, The University of Sydney, Sydney, NSW, Australia; University of Illinois at Chicago, United States of America

## Abstract

The focal adhesion docking protein NEDD9/HEF1/Cas-L regulates cell migration and cancer invasion. NEDD9 is a member of the Cas family of proteins that share conserved overall protein-protein interaction domain structure, including a substrate domain that is characterized by extensive tyrosine (Y) phosphorylation. Previous studies have suggested that phosphorylation of Y253 in the substrate domain of the Cas family protein p130Cas is specifically required for p130Cas function in cell migration. While it is clear that tyrosine phosphorylation of the NEDD9 substrate domain is similarly required for the regulation of cell motility, whether individual NEDD9 tyrosine residues have discrete function in regulating motility has not previously been reported. In the present study we have used a global sequence alignment of Cas family proteins to identify a putative NEDD9 equivalent of p130Cas Y253. We find that NEDD9 Y189 aligns with p130Cas Y253 and that it is conserved among NEDD9 vertebrate orthologues. Expression of NEDD9 in which Y189 is mutated to phenylalanine results in increased rates of cell migration and is correlated with increased disassembly of GFP.NEDD9 focal adhesions. Conversely, mutation to Y189D significantly inhibits cell migration. Our previous data has suggested that NEDD9 stabilizes focal adhesions and the present data therefore suggests that phosphorylation of Y189 NEDD9 is required for this function. These findings indicate that the individual tyrosine residues of the NEDD9 substrate domain may serve discrete functional roles. Given the important role of this protein in promoting cancer invasion, greater understanding of the function of the individual tyrosine residues is important for the future design of approaches to target NEDD9 to arrest cancer cell invasion.

## Introduction

The Cas family protein NEDD9/HEF1/Cas-L has emerged as a critical regulator of cancer invasion and metastasis in a variety of different cancers [1]. NEDD9 promotes elongated, adhesion-dependent invasion in 3D environments [2] and our previous work has suggested that NEDD9 stabilizes focal adhesions, thereby contributing to the adhesion forces that are required for the elongated mode of invasion [3]. The expression and regulation of NEDD9 is highly controlled (reviewed in [4]) and, together with other related members of the Cas family proteins, is subject to extensive phosphorylation modifications. Of particular interest is the substrate binding domain that contains multiple consensus sites for tyrosine phosphorylation [5]. Recent data have suggested that individual tyrosine residues within the substrate domain of the related protein p130Cas/BCAR1 may be associated with discrete functional outputs [6], [7]. Currently, the role of individual tyrosine residues within the substrate domain of NEDD9 is unknown.

The Cas family proteins include NEDD9, p130Cas, Efs/Sin and the most recently described member HEPL/CASS4 [8]. They are grouped together based on an overall conserved protein-protein interaction domain structure. Each of the family members contain a highly conserved N-terminal SH3 domain, followed by a less conserved substrate domain containing multiple tyrosine residues, a serine-rich region and a well-conserved C-terminal domain that has structural similarity with the Focal Adhesion Targeting (FAT) domain of FAK (reviewed in [9]). NEDD9, p130Cas and HEPL localize to focal adhesions via targeting information encompassed in both the SH3 and C-terminal FAT domains [10]–[12]. Included among prominent functions for NEDD9 and p130Cas is a role in cell migration, both in normal and pathological conditions (reviewed in [1]).

The substrate domains of both NEDD9 and p130Cas are highly tyrosine phosphorylated in response to adhesion to integrin ligands and as a result of constitutive activation of regulatory kinases including FAK and Src [4]. NEDD9 binds to FAK at focal adhesions and FAK phosphorylation of the DYDY motif in the NEDD9 c-terminus creates a binding site for Src kinase, which then catalyzes phosphorylation of tyrosines in the NEDD9 substrate domain. Phosphorylation of the NEDD9 substrate domain is required for T-lymphocyte [13], [14] and glioblastoma [15] cell migration. Similarly, the p130Cas substrate domain is required for p130Cas promotion of cell migration [16] and mutation of all 15 consensus tyrosine phosphorylation sites to phenylalanine (F) abrogates p130Cas-mediated migration [7], [17]. Y253 in the substrate domain of mouse p130Cas (equivalent to Y249 in the human sequence) was found to be the most highly phosphorylated residue by Src tyrosine kinase [6]. Correspondingly, Y to F mutation of Y253 significantly inhibited the ability of p130Cas to promote migration; although the effect of this mutation may be context dependent [7]. More recently, mutation of mouse p130Cas Y253 was shown to specifically affect migration on vitronectin and combined mutation of Y253 p130Cas together with a second site in the substrate domain were sufficient to inhibit in vivo metastasis [18]. Together, these data provide evidence that single amino acid residues in the substrate domain may regulate migration down-stream from Cas proteins.

We recently reported that mouse embryo fibroblasts (MEFs) from NEDD9−/− mice have faster rates of focal adhesion disassembly and correspondingly migrate more rapidly on 2D surfaces [3]. In the present study, we therefore questioned whether individual tyrosine residues in the NEDD9 substrate domain may play a role in regulating NEDD9-mediated cell migration and focal adhesion dynamics. Using a global sequence alignment strategy, we identified NEDD9 Y189 in alignment with p130Cas Y253 and therefore analysed the effect of mutating this residue on NEDD9 function. We show that mutation of Y189 to phenylalanine (F) to inhibit phosphorylation, results in increased rates of assembly and disassembly of NEDD9-positive focal adhesions and demonstrate that this correlates with increased rates of cell migration. Conversely, the phosphomimetic construct, Y189D, significantly inhibits cell migration. By contrast, a second NEDD9 tyrosine residue, chosen for analysis as it does not align with a corresponding tyrosine phosphorylation motif in p130Cas, had no effect on focal adhesion dynamics and cell migration.

## Results

**Figure 1:**
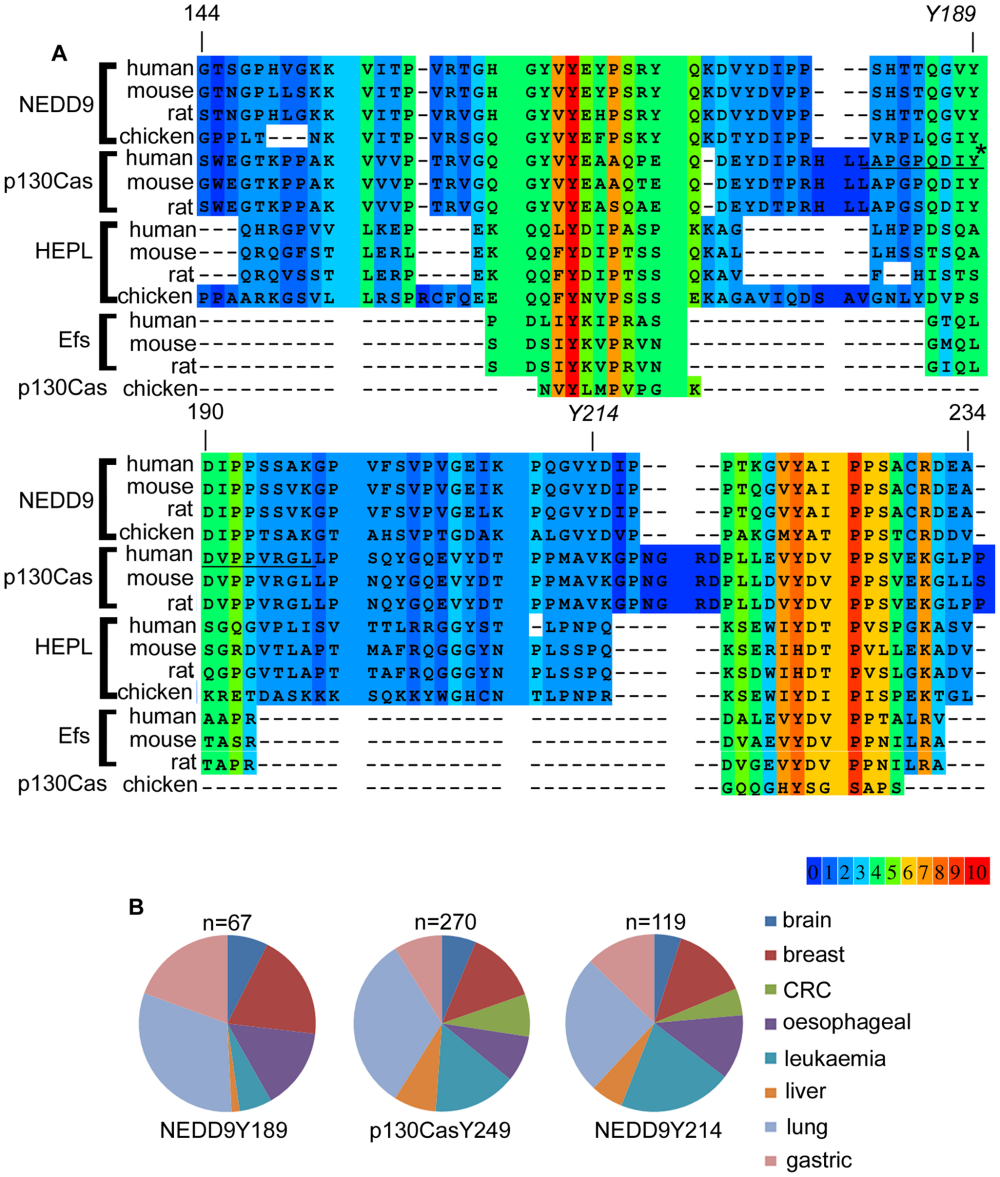
Cas family protein alignment and tyrosine phosphorylation. A. Cas family protein sequences aligned at the PRALINE website. Shown is the region of alignment encompassing human p130CasY249 (LAPGPQDIyDVPPVRGL) indicated by an asterisk and underlined. Numbers above the alignments refer to amino acid number in the NEDD9 human protein sequence, from human NEDD9 residue G144 to A234. The two highlighted tyrosine residues (*Y189*, *Y214*) indicate the two NEDD9 residues of interest. Shading indicates the degree of sequence conservation, with red (10) indicating the greatest conservation as shown in the colour gradient at the bottom of the figure. The full sequence alignment is shown in Figure S1. B. Summary of high throughput tandem mass spectrometry studies from phosphosite plus that report detection of phosphorylated NEDD9 Y189, p130Cas Y249 and NEDD9 Y214. Data accessed and analysed as per the materials and methods. Numbers (n) = the number of non-redundant positive records for each site. Pie charts show the representative distributions of the positive records between the different tumour cell types.

Given the role of mouse p130CasY253 in the regulation of p130Cas-mediated cell migration, we questioned whether there may be a functional equivalent of this residue in NEDD9. To assess potential conserved tyrosines between the Cas protein family members we performed a global sequence alignment of multiple vertebrate isoforms (human, mouse, rat and chicken) of the 4 Cas family members (Figure S1). A portion of this alignment, encompassing p130CasY253 (human p130Cas Y249), is shown (Figure 1A). The alignment reveals that p130Cas Y249 is conserved between each of the p130Cas orthologues. Moreover, Y189 of human NEDD9 aligns with human p130Cas Y249 and is conserved in all 4 vertebrate NEDD9 orthologues in the alignment (Figure 1A). Interestingly, comparison of the 13 NEDD9 tyrosine residues located in the substrate binding domain (amino acids 59–399), between the SH3 domain (amino acids 1–58) and the SRR domain (amino acids 400–462), reveals considerable concordance between the positions of NEDD9 and p130Cas tyrosines (Table 1). Of the 13 NEDD9 tyrosines, 10 show conservation with p130Cas tyrosine residues in the global alignment (although frequently the tyrosine residue is absent from the chicken p130Cas orthologue). Notably, NEDD9 Y166 and Y317 are both conserved in all Cas protein family sequences. Generally, the pattern of concordance is less between NEDD9 tryosine residues and Efs (7/13 tyrosines) and NEDD9 and HEPL (4/13 tyrosines).

**Table 1:** Conservation of tyrosine phosphorylation consensus motifs (-YXXP-) contained in Cas family protein substrate domains.

Residue #		NEDD9	P130Cas	Efs	HEPL
Y92-p	TFGQQKLyQVPNPQA	4/4	3/4	1/3	3/4
Y106-p	AAPRDTIyQVPPSyQ	4/4	–	–	–
Y118-p	SYQNQGIyQVPtGHG	4/4	3/4	3/3	–
Y132-p	GTQEQEVyQVPPSVQ	4/4	3/4	–	–
Y166-p	RTGHGYVyEYPSRYQ	4/4	4/4	3/3	4/4
Y177-p	SRVQKDVyDIPPSHT	4/4	3/4	–	–
Y189-p	SHTTQGVyDIPPSSA	4/4	3/4	–	–
Y214-p	EIKPQGVyDIPPTKG	4/4	–	–	–
Y223-p	IPPTKGVyAIPPSAC	4/4	4/4	3/3	2/4
Y241-p	AGLREKDyDFPPPMR	3/4	–	–	–
Y261-p	DLRPEGVyDIPPTCT	4/4	3/4	3/3	–
Y317-p	VGSQNDAyDVPRGVQ	4/4	3/4	3/3	4/4
Y345-p	PQERDGVyDVPLHNP	4/4	4/4	3/3	–

The amino acid residue number of the 13 NEDD9 YXXP motifs contained in the human NEDD9 substrate domain (aas 59–399) are shown in the first column. The surrounding sequence is shown in the second column. Numbers reflect conservation of the tyrosine in the orthologues examined for each Cas family protein. Human, mouse, rat and chicken orthologues were examined for all family members with the exception of Efs which did not include the chicken orthologue. Cases where no conservation was observed are indicated by a dash (–).

As an indication of whether Y189NEDD9 is phosphorylated *in vivo* we analysed publicly available data at the PhosphositePlus website. This revealed frequent detection of phosphorylated NEDD9Y189 in high throughput mass-spectrometry based screens in a variety of different cancer cell types (Figure 1B). We further compared the total number of records reporting the detection of this phosphosite with other NEDD9 residues and it was the 7^th^ most frequently detected NEDD9 phosphorylation site in the phosphosite database (detected in >100 records, Table 2). To test the hypothesis that individual tyrosine residues within the NEDD9 substrate domain may have discrete functional outcomes it was necessary to select an additional NEDD9 tyrosine to allow us to compare downstream phenotypes. Thus, for this purpose we chose NEDD9 Y214 (detected in >200 records, Table 2) that displayed no alignment with tyrosines in any of the other Cas proteins and therefore might be expected to serve a NEDD9-specific function. Analysis of phosphopeptide data at PhosphositePlus also confirmed frequent detection of phosphorylated human p130Cas Y249 (detected in >600 records, Table 3) and NEDD9 Y214 in a variety of cancer cell lines (Figure 1B). These data therefore provide evidence that these sites may be bona fide phosphorylation targets *in vivo*.

**Table 2:** Individual NEDD9 residue phosphorylation frequency.

NEDD9		
# records	Residue	Phospho-peptide
604	Y317	VGsQNDAyDVPRGVQ
491	Y166	RtGHGyVyEyPSRyQ
307	Y345	PQERDGVyDVPLHNP
271	Y92	TFGQQKLyQVPNPQA
205	Y241	AGLREKDyDFPPPMR
204	Y214	EIKPQGVyDIPPtKG
110	Y189	sHttQGVyDIPPSSA
69	Y177	SRyQKDVyDIPPsHt
60	Y629	ERSWMDDyDyVHLQG
47	Y631	SWMDDyDyVHLQGKE
25	Y261	DLRPEGVyDIPPTCT
20	Y164	PVRtGHGyVyEyPSR
12	Y132	GtQEQEVyQVPPSVQ
11	Y106	AAPRDTIyQVPPSyQ
11	S182	DVyDIPPsHttQGVy
10	Y223	IPPtKGVyAIPPsAC
7	Y118	SyQNQGIyQVPtGHG
7	Y168	GHGyVyEyPSRyQKD
7	Y172	VyEyPSRyQKDVyDI
5	T219	GVyDIPPtKGVyAIP

Ranked list showing numbers of mass spectrometry records of phosphorylation at the indicated residue. Phospho-sites with <5 records are not included. Data from Phophositeplus (www.phosphosite.org).

**Table 3:** Individual p130Cas residue phosphorylation frequency.

p130Cas		
# records	Residue	Phospho-peptide
662	Y128	SKAQQGLyQVPGPsP
607	Y249	APGPQDIyDVPPVRG
603	Y234	AQPEQDEyDIPRHLL
382	Y387	RPGPGtLyDVPRERV
272	Y287	RDPLLEVyDVPPsVE
255	Y267	SQyGQEVyDtPPMAV
192	Y327	PLLREEtyDVPPAFA
146	Y306	PsNHHAVyDVPPsVs
86	Y224	RVGQGyVyEAAQPEQ
85	Y410	GVVDSGVyAVPPPAE
61	T385	LRRPGPGtLyDVPRE
52	Y372	PPPAPDLyDVPPGLR
43	Y362	sPPAEDVyDVPPPAP
22	Y666	GWMEDyDyVHLQGKE
21	Y664	EGGWMEDyDyVHLQG
17	T326	GPLLREEtyDVPPAF
12	Y115	QPQPDSVyLVPTPSK
12	T269	yGQEVyDtPPMAVKG
11	S139	GPsPQFQsPPAkQTS
8	H552	LQKMEDVHQTLVAHG
6	Y222	PTRVGQGyVyEAAQP
5	Y192	AGMGHDIyQVPPSMD
5	S300	VEKGLPPsNHHAVyD

Ranked list showing numbers of mass spectrometry records of phosphorylation at the indicated residue. Phospho-sites with <5 records are not included. Data from Phophositeplus (www.phosphosite.org).

**Figure 2:**
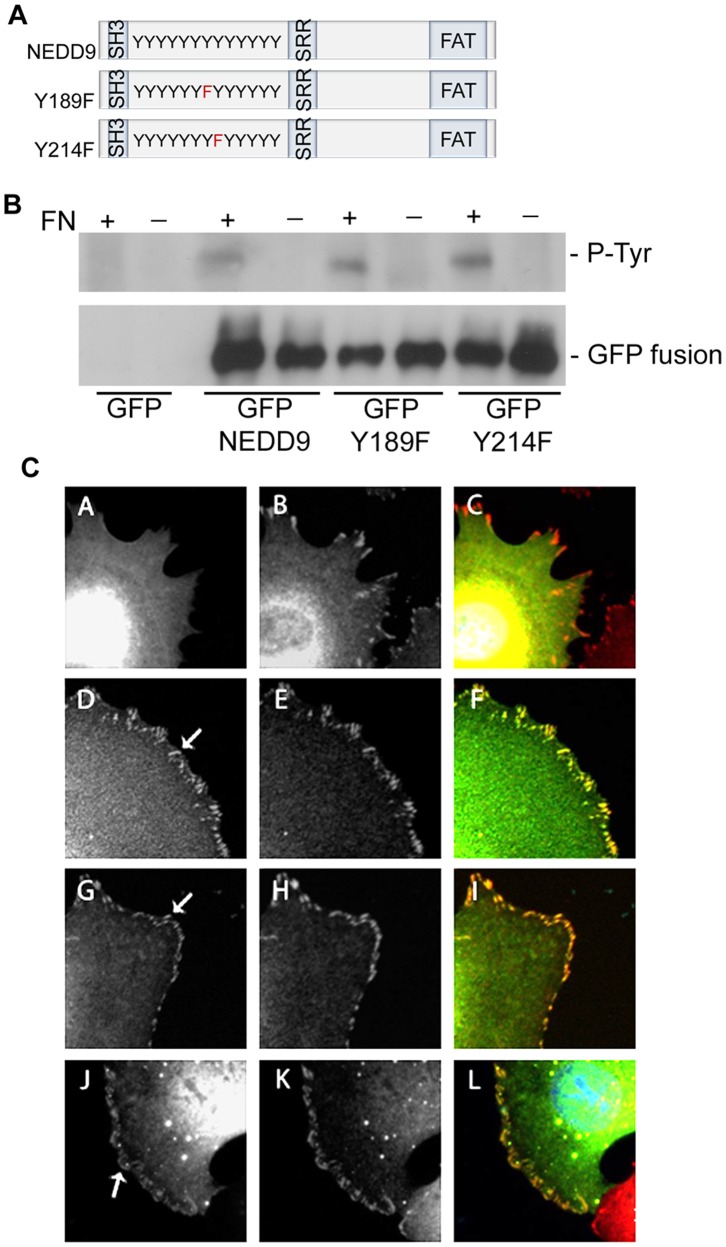
NEDD9 mutations do not affect global tyrosine phosphorylation or targeting to focal adhesions. A. Schematic representation of NEDD9 protein sequence showing the N-terminal SH3 domain, substrate domain containing 13 consensus tyrosine phosphorylation motifs (Y), the serine rich domain (SRR) and the Focal Adhesion Targeting domain (FAT). The two mutated tyrosine residues are indicated in red in the Y189F and Y214F schematic representations. B. Cells transfected with the indicated expression constructs were held in suspension (–) or suspended and then plated onto fibronectin (+). NEDD9 immunoprecipitates were probed with antibodies to phosphotyrosine (P-Tyr) and with anti-NEDD9 antibodies (GFP fusions). C. Cells transfected with GFP vector (A–C), GFP.NEDD9 (D–F), GFP.NEDD9 Y189F (G–I) and GFP.NEDD9 Y214F (J–L). Left hand panels show GFP fluorescence images, middle panels show paxillin immunostaining and merged images are shown on the right. Arrows point to examples of positive focal adhesions. Each image shows a cropped region of one cell.

To analyse the effect of the individual tyrosines, NEDD9 expression constructs were generated in which either Y189 or Y214 were mutated to phenylalanine (Y189F and Y214F) to prevent phosphorylation (Figure 2A). The effect of the individual mutations on total NEDD9 phosphorylation was determined by plating cells in suspension (FN-) followed by plating on fibronectin (+) to stimulate adhesion and corresponding tyrosine phosphorylation. Similar levels of tyrosine phosphorylation were induced by adhesion in either exogenously expressed mutant proteins and in the wild-type NEDD9 exogenous control (Figure 2B), reflecting the large number of tyrosines in the substrate domain. Moreover, comparison of the sub-cellular distribution of GFP-tagged NEDD9 fusion proteins with paxillin-positive focal adhesions revealed that both NEDD9Y189F and NEDD9Y214F are efficiently targeted to focal adhesions (Figure 2C). Therefore, mutation of the either tyrosine residues does not appear to block global adhesion-dependent NEDD9 tyrosine phosphorylation and neither are they required for NEDD9 localization to focal adhesions.

**Figure 3:**
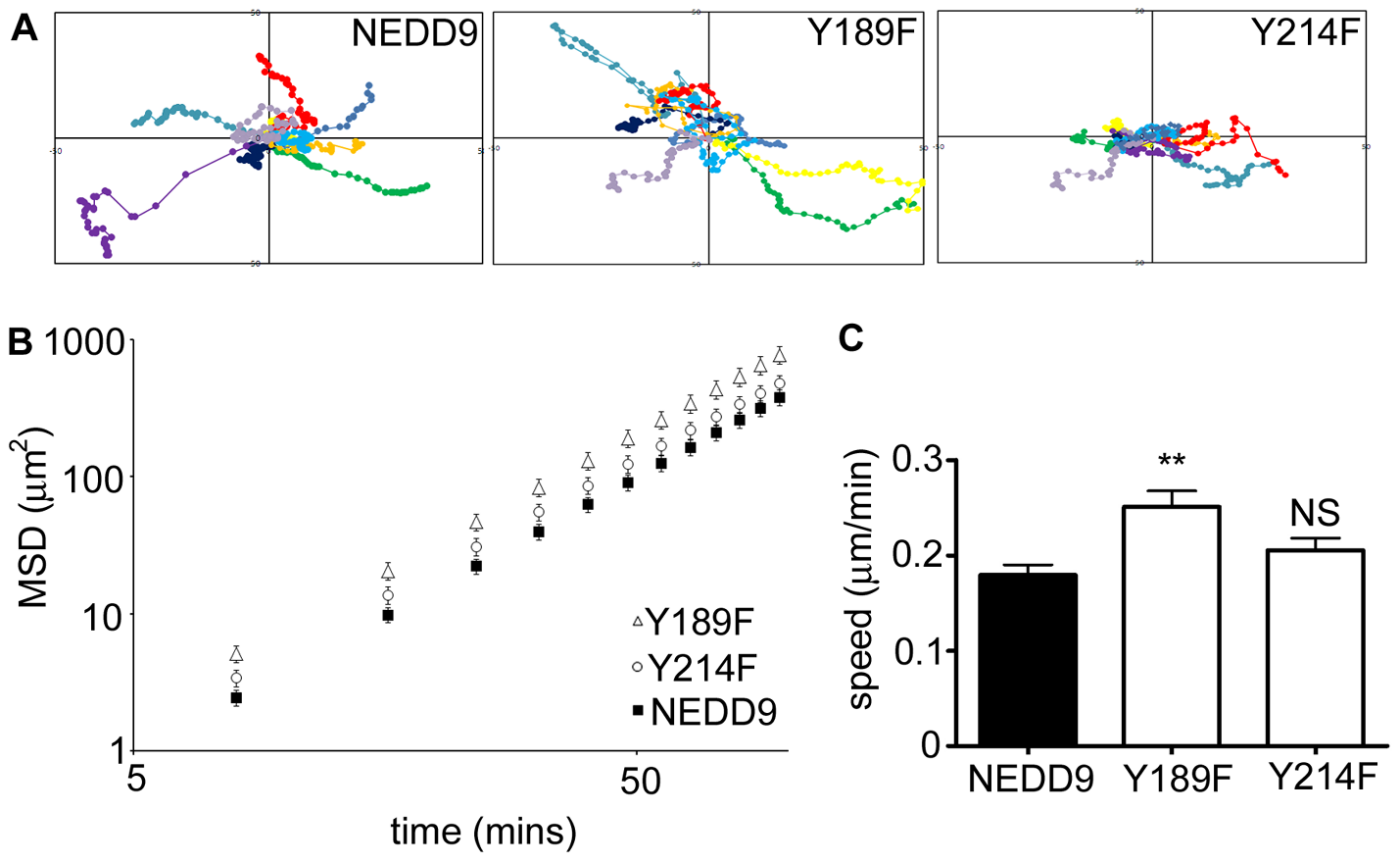
Mutation of NEDD9Y189 stimulates faster cell migration. A. 10 representative migration traces of NEDD9−/− fibroblast cells transfected with the indicated GFP expression plasmids. B. MSD calculated from trajectories of cells expressing exogenous NEDD9 (black squares), NEDD9 Y189F (white triangles) and Y214F (white circles). C. Average speed of NEDD9−/− fibroblasts transfected with the indicated GFP expression plasmids. Graphs show the average (n>50 cells per expression construct) and SEM. **p<0.01, NS = not significant.

Next, we analysed the role of NEDD9 Y189 and NEDD9 Y214 in the promotion of cell migration. NEDD9−/− mouse embryo fibroblasts were transfected with pGFP.NEDD9, pGFP.NEDD9 Y189F and pGFP.NEDD9 Y214F and individual migrating cells were imaged by time-lapse microscopy. Analysis of the migration paths traced by the cells indicated that cells expressing NEDD9 Y189F elaborated extensive migration paths (Figure 3A). Quantification of the Mean Squared Displacement (MSD) revealed that the cells expressing NEDD9 Y189F have significantly faster migration speed than cells expressing wild-type exogenous NEDD9 (Figure 3B and 3C). By contrast, migration paths of cells expressing NEDD9 Y214F were less extensive (Figure 3A) and quantification of MSD for these cells confirmed that their speed is not significantly different to control cells transfected with wild-type NEDD9 (Figure 3B and 3C).

**Figure 4:**
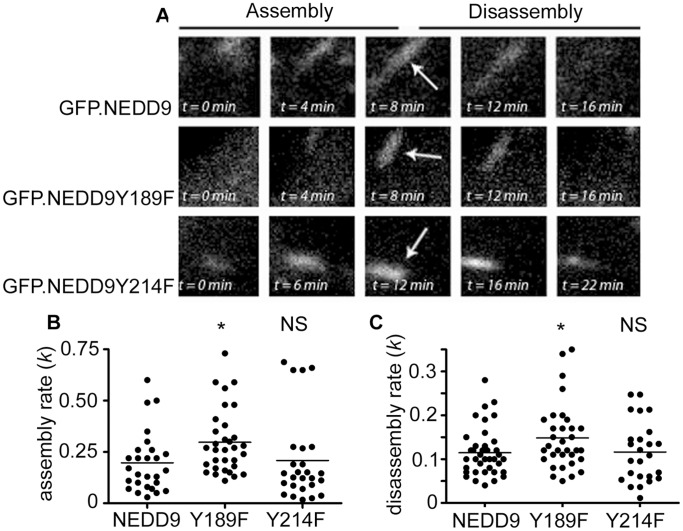
Mutation of NEDD9Y189 stimulates faster adhesion dynamics. A. Time-lapse microscopy of the assembly and disassembly of GFP-positive focal adhesions. Shown are examples of focal adhesions in NEDD9−/− MEFs transfected with GFP.NEDD9, GFP.NEDD9Y189F and GFP.NEDD9 Y214F. Each box shows a 4.7 µm×4.7 µm cropped region. Arrows point to each adhesion at the time of peak fluorescence intensity. B. Focal adhesion assembly rate constants (*k*) for the indicated fusion proteins. C. As for B, except data showing the disassembly rate constants (*k*). N>25 individual adhesions were analysed per condition. *p<0.05, N.S. = not significant.

Having previously demonstrated that NEDD9 expression regulates focal adhesion dynamics [3] we hypothesized that faster cell migration speeds induced by expression of NEDD9 Y189F may reflect faster focal adhesion turnover induced by this mutation. Therefore, we analysed GFP.NEDD9 Y189F positive focal adhesions in NEDD9−/− MEFs by time-lapse microscopy (Figure 4A). Quantification indicates that GFP.NEDD9Y189F-positive adhesions assemble more rapidly than wild-type GFP.NEDD9 adhesions (Figure 4B). Similarly, they disassembled more rapidly than their wild-type counterparts (Figure 4C). In contrast, GFP.NEDD9 Y214F positive adhesions have the same rates of assembly and disassembly as GFP.NEDD9 adhesions (Figure 4B and 4C). Thus the faster migration speed stimulated by GFP.NEDD9 Y189F expression is correlated with increased assembly and disassembly of GFP.NEDD9 Y189F focal adhesions. Together, these data suggest that phosphorylation of NEDD9 Y189F may be critical to NEDD9 regulation of focal adhesion dynamics and cell migration speed.

**Figure 5:**
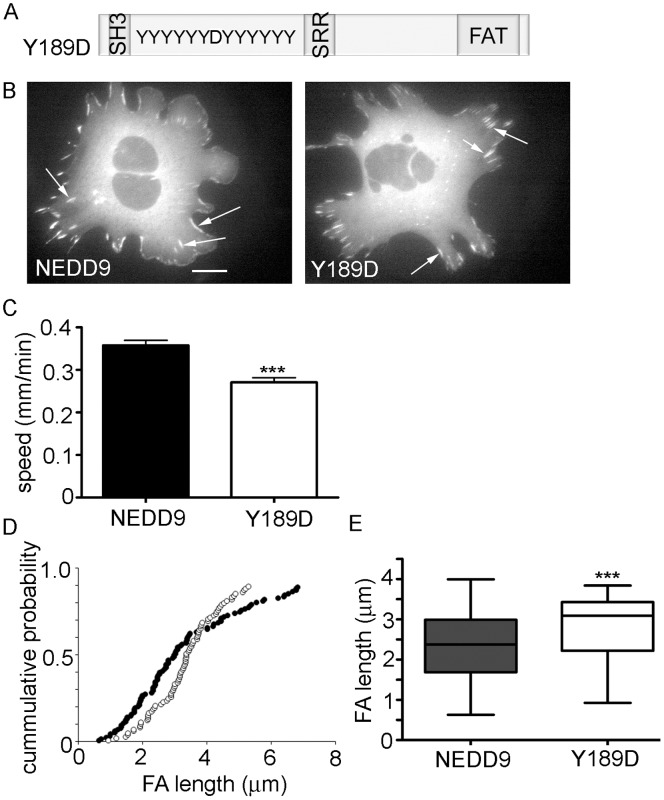
NEDD9Y189D phospho-mimetic inhibits migration and increase focal adhesion lengths. A. Schematic representation of NEDD9 protein sequence showing the Y189D mutation. B. NEDD9−/− MEFs transfected with GFP.NEDD9 or GFP.NEDD9Y189D, as indicated. Arrows point to examples of positive focal adhesions. Scale bar = 20 µm. C. Average speed of NEDD9−/− fibroblasts transfected with the indicated GFP expression plasmids. Graphs show the average (n>100 cells per expression construct) and SEM. ***p<0.001. D. Fraction of focal adhesions versus adhesion length. Data for GFP.NEDD9 show in black circles (n = 112) and for GFP.Y189D in white circles (n = 96). E. Comparison of the fraction of focal adhesions less than 4 µm long. ***p<0.001.

To next confirm whether phosphorylation of Y189 is sufficient to regulate cell migration speed, we tested the effect of the phospho-mimetic, Y189D (Figure 5A). Importantly, this mutation did not affect NEDD9 targeting to focal adhesions (Figure 5B). Time-lapse imaging of transfected cells, followed by cell tracking revealed that indeed this mutation significantly reduced cell migration speed, when compared with cells transfected with wild-type GFP.NEDD9 (Figure 5C). Given our data showing that NEDD9Y189F-positive focal adhesions had more rapid dynamics than their wild-type counterparts, we questioned whether the phospho-mimetic GFP.NEDD9Y189D positive focal adhesions may have reduced dynamics when compared with wild-type GFP.NEDD9 adhesions. Careful analysis of the focal adhesions after time-lapse imaging failed to reveal a change in the dynamics of GFP.NEDD9Y189D-positive adhesions. However, it was noted that the GFP.NEDD9Y189D focal adhesions appeared to be more uniform in size than their wild-type counterparts. Thus we compared focal adhesion lengths in regions of protruding cell membrane. This analysis revealed an apparent dichotomy in the focal adhesion size, with a greater frequency of longer GFP.NEDD9Y189D less that 4 µm long and conversely a greater frequency of longer GFP.NEDD9 focal adhesions greater than 4 µm (Figure 5D). Indeed, comparison of the focal adhesions less than 4 µm long, revealed that the GFP.NEDDY189D focal adhesions are significantly longer in this fraction of the population (Figure 5E). Thus the data suggest that GFP.NEDD9Y189D focal adhesions are more tightly clustered around lengths of 4 µm.

## Discussion

Previous studies have suggested an important role for phosphorylation of Y253 mouse/Y249 human p130Cas in the regulation of cell migration. Using a global sequence alignment strategy we identified that Y189 in the human NEDD9 substrate domain appears to be conserved with Y249 of human p130Cas. We further show that NEDD9 Y189 appears to contribute to focal adhesion dynamics and cell migration. Thus our study has identified a unique role for a single amino acid residue in the substrate domain of NEDD9. Collectively, our data and previously published investigations of p130Cas tyrosine residues [6], [18] suggest that individual tyrosine phosphorylation events in the Cas protein substrate domains confer discrete functional outcomes.

The global alignment of the Cas family sequences revealed a striking consensus between NEDD9 and p130Cas tyrosine phosphorylation motifs (-YXXP-) in the substrate domains. NEDD9 Y189 is one of 10 tyrosines that align with p130Cas tyrosines and only 3 NEDD9 tyrosines (including Y214) show no concordance. Interestingly, two tyrosines – NEDD9 Y166 and NEDD9 Y317 - showed concordance in all 4 Cas proteins. The p130Cas mouse tyrosine Y391 that aligns with NEDD9 Y317 has previously been shown to be a substrate for Src kinase [6]. In addition to the tyrosines, we note conservation of serine (S) 369 NEDD9 in the p130Cas and HEPL human, mouse and rat sequences. Previous studies have shown that this residue is critical for NEDD9 targeting by the proteasome [19] and plays a role in NEDD9-mediated cell spreading [20]. Leucine (L) 751 of NEDD9 that mediates binding to BCAR3/AND34/NSP2 [21] is conserved in p130Cas and this site has recently been confirmed to mediate the same interaction for p130Cas [22]. By contrast S296 that is phosphorylated by Aurora kinase [23] is unique to the NEDD9 sequence. Thus the Cas family proteins appear to have both overlapping and distinct site specific functions.

We have previously shown that mouse embryo fibroblasts (MEFs) from NEDD9−/− mice have faster rates of focal adhesion disassembly and correspondingly migrate more rapidly on 2D surfaces [3]. Consequently, this indicates that NEDD9 may function to stabilize focal adhesions and thus regulate the rate of cell migration. In the present study we show that mutation of NEDD9 Y189 to disable phosphorylation of this residue both increases cell migration speed. Conversely, the phosphomimetic form, Y189D, significantly reduced cell migration speed. This therefore suggests that Y189 may be required for NEDD9 stabilization of focal adhesions. The goal of our study was to determine whether there is a functional equivalent of p130Cas Y253 in the sequence of NEDD9 and we have indeed identified that Y189 NEDD9 plays a specific role in NEDD9 stimulation of cell migration. However, the two residues appear to play opposing roles in regulating the migration phenotypes of their respective proteins. Thus, while NEDD9 Y189F results in increased migration relative to cells transfected with wild-type NEDD9, p130Cas Y253F inhibited migration relative to cells expressing wild-type p130Cas [6]. This may reflect different effects between the two molecules on the rate of focal adhesion turnover. Previous studies in MEFs have suggested that p130Cas expression induces increased rates of focal adhesion turnover [24], [25]. In contrast our previous data have suggested that NEDD9 stabilizes focal adhesions in MEFs [3]. Therefore the differences in effect between Y253 p130Cas and Y189 NEDD9 are congruent with these differential roles in adhesion stability. Currently, it is not known what mediates this differential effect on focal adhesion turnover. However, it is clear that despite the high similarity between the substrate binding domains of these two proteins, they respond differently to stimuli that induce tyrosine phosphorylation [15], [26] and mediate differential down-stream signalling pathway activation [2], [27].

At present the protein(s) that associate with phosphorylated Y189 NEDD9 are not known. Our data suggests that mutation of this residue does not affect global tyrosine phosphorylation of NEDD9. Therefore, it appears likely that phosphorylated Y189 may mediate interaction with a specific protein partner that is required for NEDD9 stabilization of focal adhesions. Conceptually, the abundance of phosphorylation sites in both the p130Cas/BCAR1 and NEDD9 substrate binding domains suggests the possibility of redundancy among these sites. However, together with our study, evidence is now mounting to suggest that at least some individual sites may be necessary for discrete functions of each molecule. Given the critical role of NEDD9 in the promotion of cancer invasion [1], elucidation of NEDD9-specific and NEDD9-unique phosphorylation-mediated interactions is likely to be important for future assessment of NEDD9 as both a prognostic indicator and therapy target in cancer.

## Materials and Methods

### Sequence Alignments and Phospho-peptide Data Analysis

Global sequence alignment was performed using PRALINE (http://www.ibi.vu.nl/programs/pralinewww/) [28]. The following sequences were compared: NEDD9 *Homo sapiens* (AAH20686), *Mus musculus* (NM_017464), *Rattus norvegicus* (NM_001011922), *Gallus gallus* (XM_418946); p130Cas *Homo sapiens* (P56945), *Mus musculus* (NM_009954), *Rattus norvegicus* (NM_012931), *Gallus gallus* (XM_414057); Efs *Homo sapiens* (NP_005855.1), *Mus musculus* (AAC52340.1), *Rattus norvegicus* (AAI61942); and HEPL *Homo sapiens* (Q9NQ75), *Mus musculus* (AAI29977.1), *Rattus norvegicus* (NP_001178673.1), *Gallus gallus* (XP_417499.2). Curated, publicly available, high-throughput tandem mass spectrometry-derived phosphorylation modification data from Phophositeplus (www.phosphosite.org) [29] were analysed. Analysis was restricted to Cell Signalling Technology curation set data and records were manually screened for duplications (for example the same data set appearing under separate categories of brain cancer and glioblastoma). Records were grouped as brain (brain, glioblastoma and glioma), breast (breast cancer, breast adenocarcinoma, breast ductal carcinoma, breast cancer triple-negative), colorectal, gastric, leukaemia (acute myeloid, chronic myeloid, acute lymphocytic and chronic lymphocytic), liver and lung (lung, non-small cell lung, non-small-cell squamous cell lung, small-cell lung) cancers. Tumour cell types in which <5 examples were detected for at least one of the phospho-tyrosine residues were excluded from the analysis.

### Cell Culture and Antibodies

Immortalized mouse embryo fibroblasts (MEF) from homozygous null NEDD9 (NEDD9−/−) mice have been previously described [3]. MEFs and MCF-7 breast cancer cells (as previously used [31]) were maintained in Dulbecco's Modified Eagles Medium (DMEM) (Invitrogen) with 15% and 10% Foetal Bovine Serum (FBS), respectively. MEF media was supplemented with antibiotics (pen/strep, Invitrogen). Monoclonal antibodies to NEDD9 (clone 2G9) were from ImmuQuest (Cleveland, UK), monoclonal anti-phosphotyrosine antibody (clone 4G10) was purchased from Upstate Millipore (CA, USA), anti-paxillin antibody was from BD Transduction Laboratories (NW, USA). Secondary HRP-conjugated anti-mouse and anti-rabbit antibodies were either from Amersham (NJ, USA) and Cy3-conjugated anti-mouse antibodies were purchased from Jackson Immunoresearch (PA, USA).

### Expression Constructs and Transfections

The GFP.NEDD9 fusion protein expression construct has been previously described (Law et al., 2000) and was used as the template to create single amino acid substitutions of tyrosine (Y) to phenylalanine (F) by site-directed mutagenesis using the Quikchange mutagenesis kit from Stratagene (La Jolla, CA). The forward primer sequence to generate Y189F was 5′-GGGGTATTTGACATCCCTCCCTCATCAGCAAAAGGC-3′ and to generate Y214F was 5′-GATAAAACCTCAAGGGGTGTTTGAGCATCCCGCCTACAAAAGGG-3′. Forward primers and the matched reverse complementary primers were purchased from Sigma (USA). Generation of the Y189D mutant construct was performed by Genscript (NJ, USA). All cDNA constructs were sequenced in both directions to confirm sequence fidelity. Transient transfection of MEF cells was achieved using a nucleofector (Amaxa) and MEF2 nucleofector kit, as per the manufacturer’s protocol (Integrated Sciences). MCF-7 cells were transfected using Lipofectamine 2000 as per the manufacturer’s instructions (Invitrogen Corporation, CA, USA).

### Protein Extraction, Immunoblotting, Cell Suspension and Immunoprecipitation

Conditions of protein extraction and immunoblotting were carried out as previously described [30]. Adhesion to fibronectin to stimulate NEDD9 tyrosine phosphorylation was achieved essentially as previously described [31], [32]. Briefly, after transfection, MCF-7 cells were serum starved overnight, and the following day cells were detached by 15 minutes incubation with 3 mM EDTA in PBS at 37°C. Cells were resuspended in serum-free DMEM and incubated on bacterial petri dishes (to prevent adhesion) for 2 hours. Cells were then either extracted after 2 hours (suspension conditions) or replated onto fibronectin coated dishes in serum-free media for 2 hours. Proteins from cells grown in suspension or adhered to fibronectin were extracted in NP-40 lysis buffer (10 mM Tris-HCL pH7.5, 5 mM EDTA, 50 mM NaF, 1% v/v Nonidet P-40, 0.05% w/v SDS and 0.25% v/v sodium deoxycholate; extraction buffer freshly supplemented with 2 mM Na_3_VO_4_, 1 mM PMSF, 10 µg/ml Leupeptin, 10 µg/ml Aprotinin immediately prior to extraction). Extracted NEDD9 proteins were immunoprecipitated with anti-NEDD9 antibodies coupled to protein A Sepharose (Sigma, MO, USA).

### Live and Fixed Cell Imaging

Cells were grown in 35 mm glass bottom dishes (MatTek) in recording media (CO_2_-independent media (Invitrogen), supplemented with 15% FBS and pen-strep). Immediately prior to imaging, recording media was supplemented with Oxyrase (Oxyrase, Ohio, USA) (60 µl per 2 mL media). Time-lapse images were captured using an ORCA ERG cooled CCD camera (Hamamatsu, SDR Clinical Technology NSW, Australia) and Olmypus IX81 inverted microscope equipped with an environmental chamber heated to 37°C. For assay of focal adhesion dynamics and random cell motility glass bottom dishes were pre-treated with poly-L-lysine (50 µg/mL) and then coated with a solution of fibronectin (20 µg/mL) for 2 hours at 37°C. For fixed imaging, cells were fixed in 4% PFA in PBS for 10 minutes, permeabilised (0.2% v/v Triton X-100, 0.5% w/v BSA in PBS) and then immunostained. Imaging of fixed cells was performed with the Olympus IX81 inverted microscope.

### Migration Analysis

Transmitted light images of MEF cells, or fluorescence images of GFP-transfected MEF cells, were captured every 8 minutes for 160 mins, (40× objective). Cells undergoing division or apoptosis were excluded from analyses and random migration analyses were performed on sparsely plated cultures. Post image capture, nuclear translocation was tracked in time-lapse stacks using Metamorph V6.3 software (Molecular Devices). Generation of migration traces and the calculation Mean Squared Displacement and cell speeds were performed as previously described [3]. Measurements were performed with Metamorph V6.3 software, calculations were performed in Microsoft Excel and graphs generated in GraphPad Prism.

### Measurement of Focal Adhesion Dynamics and Lengths

GFP-NEDD9-positive focal adhesions were imaged with an Olympus IX81 inverted microscope, with a 60X (NA 1.35) oil objective, using fluorescence filters of BP 460–495/BP510–550 (GFP). Images were captured every 2 minutes for 90 minutes total, 250 ms exposure times. Focal adhesions at the protruding edge of the membrane were analysed by measuring the temporal changes in integrated pixel intensity of an individual focal adhesion. Quantification of adhesion dynamics was performed as previously described [3] with data from >25 adhesions pooled from each of 3–5 cells per construct. Lengths of focal adhesions at the protruding edge were measured on calibrated images.

### Image Preparation

Final micrograph images and grey level adjustments were prepared in Adobe Photoshop.

### Statistical Analysis

All error bars on histograms show the standard error of the mean (SEM). Statistical comparison of two means was performed in Graph Pad Prism using a Student’s *t* test and for greater than two means using one-way ANOVA with Tukey's Multiple Comparison Test.

## Supporting Information

Figure S1 **Cas family proteins sequence alignment.** Sequences aligned at the PRALINE website.
(PDF)
