# Supplementary material for: Tyrosine Y189 in the Substrate Domain of the Adhesion Docking Protein NEDD9 Is Conserved with p130Cas Y253 and Regulates NEDD9-Mediated Migration and Focal Adhesion Dynamics
Source: PLoS One. 2013 Jul 9;8(7):e69304. doi: 10.1371/journal.pone.0069304 (PMC3706375; doi:10.1371/journal.pone.0069304)
Supplement: Figure S1 — Cas family proteins sequence alignment. Sequences aligned at the PRALINE website. (PDF) [file pone.0069304.s001.pdf]

Results colour-coded for amino acid conservation

The current colourscheme of the alignment is for **amino acid conservation**.

The conservation scoring is performed by PRALINE. The scoring scheme works from 0 for the least conserved alignment position, up to 10 for the most conserved alignment position. The colour assignments are:

Unconserved 0 1 2 3 4 5 6 7 8 9 10 **Conserved**

```
            .........10 .........20 .........30 .........40 .........50
NEDD9_Hs    ----------  ----------  ----------  ----------  ----------
NEDD9_Mm    ----------  ----------  ----------  ----------  ----------
NEDD9_Rn    ----------  ----------  ----------  ----------  ----------
NEDD9_Gg    ----------  ----------  ----------  ----------  ------MKYK
p130Cas_Hs  ----------  ----------  ----------  ----------  ----------
p130Cas_Mm  ----------  ----------  ----------  ----------  ----------
p130Cas_Rn  MKYLVSVGAG  PARRAGGLED  VSWGPRVSRR  PQSYRAARHV  NESLPRSAFR
HEPL_Hs     ----------  ----------  ----------  ----------  ----------
HEPL_Mm     ----------  ----------  ----------  ----------  ----------
HEPL_Rn     ----------  ----------  ----------  ----------  ----------
HEPL_Gg     ----------  ----------  ----------  ----------  ----------
Efs_Hs      ----------  ----------  ----------  ----------  ----------
Efs_Mm      ----------  ----------  ----------  ----------  ----------
Efs_Rn      ----------  ----------  ----------  ----------  ----------
p130Cas_Gg  ----------  ----------  ----------  ----------  ----------
Consistency 0000000000  0000000000  0000000000  0000000000  0000000000

            .........60 .........70 .........80 .........90 .........100
NEDD9_Hs    ----------  ----------  ----------  ----------  ----MKYKNL
NEDD9_Mm    ----------  ----------  ----------  ----------  ----MKYKNL
NEDD9_Rn    ----------  ----------  ----------  ----------  ----MKYKNL
NEDD9_Gg    VSGGGGDGDG  ----------  ------LCFQ  QSLLAAGTGL  R-VWCLRQNL
p130Cas_Hs  ----------  ----------  ----------  ----------  ----MNHLNV
p130Cas_Mm  ----------  ----------  ----------  ----------  ----MTVPNV
p130Cas_Rn  VPAAHGASVT  PSAALGSGLP  ETQPEAVCRG  TEKPRFAEGC  KPAASRDKNV
HEPL_Hs     ----------  ----------  ----------  ------MKGT  GIMDCAPKAL
HEPL_Mm     ----------  ----------  ----------  ------MRGT  SIREGAPKTL
HEPL_Rn     ----------  ----------  ----------  ------MRGT  GIREGATKTL
HEPL_Gg     ----------  ----------  ----------  ----------  ----MKVNNA
Efs_Hs      ----------  ----------  ----------  ----------  --MAIATSTQ
Efs_Mm      ----------  ----------  ----------  ----------  --MAIATSAQ
Efs_Rn      ----------  ----------  ----------  ----------  --MAIATSAQ
p130Cas_Gg  ----------  ----------  ----------  ----------  -------XNV
Consistency 0000000000  0000000000  0000000000  0000000000  0011343-21  4748364865

            .........110 .........120 .........130 .........140 .........150
NEDD9_Hs    MARALYDNVP  ECAEELAFRK  GDILTVIEQN  -TGGLEGWWL  CSLHGRQGIV
NEDD9_Mm    MARALYDNVP  ECAEELAFRK  GDILTVIEQN  -TGGLEGWWL  CSLHGRQGIV
NEDD9_Rn    MARALYDNVP  ECAEELAFRK  GDILTVIEQN  -TGGLEGWWL  CSLHGRQGIV
NEDD9_Gg    MARALYDNVP  ECAEELAFRK  GDILTVIEQN  -TEGLEGWWL  CSLHGRQGIV
p130Cas_Hs  LAKALYDNVA  ESPDELSFRK  GDIMTVLEQD  -TQGLDGWWL  CSLHGRQGIV
p130Cas_Mm  LAKALYDNVA  ESPDELSFRK  GDIMTVLERD  -TQGLDGWWL  CSLHGRQGIV
p130Cas_Rn  LAKALYDNVA  ESPDELSFRK  GDIMTVLERD  -TQGLDGWWL  CSLHGRQGIV
HEPL_Hs     LARALYDNCP  DCSDELAFSR  GDILTILEQH  -VPESEGWWK  CLLHGRQGLA
HEPL_Mm     LARALYDNHA  DCSDELAFSR  GDILTIVEQN  -VPESEGWWR  CLLHGRQGLA
HEPL_Rn     LARALYDNHP  DCSDELAFSR  GDILTIVEQN  -VPESEGWWK  CLLHGRQGLA
HEPL_Gg     LAKALYDNKA  ECSDELAFRR  GDILTVLEQH  -VVGSEGWWR  CSLHGRQGLA
Efs_Hs      LARALYDNTA  ESPQELSFRR  GDVLRVLQRE  GAGGLDGWCL  CSLHGQQGIV
Efs_Mm      LARALYDNTA  ESPQELSFRR  GDVLRVLQRE  GAGGLDGWCL  CSLHGQQGIV
Efs_Rn      LARALYDNTA  ESPQELSFRR  GDVLRVLQRE  GAGGLDGWCL  CSLHGQQGIV
p130Cas_Gg  LAKALYDNVA  ESPDELSFRK  GDIMTVLERN  -TQGLDGWWL  CSLHGRQGIV
Consistency 8*8*****56  8657**8*78  **98798876  064768**76  *7***8**87

            .........160 .........170 .........180 .........190 .........200
NEDD9_Hs    PGNRVKLLIG  PMQET-----  ----------  ----------  ----------
NEDD9_Mm    PGNRVKLLIG  PVQET-----  ----------  ----------  ----------
NEDD9_Rn    PGNRVKLLIG  PVQEP-----  ----------  ----------  ----------
NEDD9_Gg    PGNRVKLLIG  PVVQD-----  ----------  ----------  ----------
p130Cas_Hs  PGNRLKILVG  MYDKKPAGPG  SGPPATPAQP  QP----GLHA  PAPPASQYTP
p130Cas_Mm  PGNRLKILVG  MYDKKPVGPG  PGPPATPPQP  QPSLPQGVHA  PVPPASQYSP
p130Cas_Rn  PGNRLKILVG  MYDKKPAAPG  PGPPATPPQP  QPSLPQGVHT  PVPPASQYSP
HEPL_Hs     PANRLQILTE  VAADRPCPPF  LRGLEEAPAS  SE-------E  TYQVPTLPRP
HEPL_Mm     PANRLQVLRE  TPADRPCPLL  PRGPDTDLTS  SG-------A  PYQVQDLISP
HEPL_Rn     PANRLQVLRE  TPVDRPCPLP  PRGLDSDLTG  SE-------A  PCQVFNSFSP
```

```
HEPL_Gg     PANRLQLLAG -PQALPPPAI PSDAPEPPVA PQ-------N IYQVPSVPKG
Efs_Hs      PANRVKLLPA GPAPK----- ---------- ---------- ----------
Efs_Mm      PANRVKLLPA GPAPK----- ---------- ---------- ----------
Efs_Rn      PANRVKLLPA SPAPK----- ---------- ---------- ----------
p130Cas_Gg  PGNRLKILVG MYDKK----- ---------- ---------- ----------
Consistency *7**888*45 3444520100 1000011000 1000000001 1011010011

            .........210.........220.........230.........240.........250
NEDD9_Hs    -----ASSHE QPASGLMQQT FGQQKLYQVP NPQAAPRDTI YQVPP-----
NEDD9_Mm    -----PG-HE QPTPGPMHQT FGQQKLYQVP NSQAASRDTI YQVPP-----
NEDD9_Rn    -----SS-HE QPTPGPMHQN FGQQKLYQVP NSQAAPRDTI YQVPP-----
NEDD9_Gg    ----GPSSQD MSNSGLTHQS FNQQKIYQVP SSHASARDPV YQVPP-----
p130Cas_Hs  MLPNTYQPQP DSVYLVPTPS KAQQGLYQVP GPSPQFQSPP AKQTSTFSKQ
p130Cas_Mm  MLPTAYQPQS DNVYLVPTPS KTQQGLYQAP GPNPQFQSPP AKQTSTFSKQ
p130Cas_Rn  MLPTAYQPQP DNVYLVPTPS KTQQGLYQAP GPNPQFQSPP AKQTSTFSKQ
HEPL_Hs     PTPGPVYEQM RSWVEGPQPP T--AQVYEFP DPPTSARIIC EKTL------
HEPL_Mm     PPQGPVYEPM RSWVEGPSPA T--AQVYELP ESPSSARIIC EKTL------
HEPL_Rn     PTPGPVYEPM KSWVEGPSPA T--AQVYELP DSPSSARIIC EKTL------
HEPL_Gg     TASSSTYEKM EGWVTSPARA ATLPAQGVYQ VPALAAQLLS ERTR------
Efs_Hs      ---------- -PSLSPASPA QPG-SPYPAP DHSN------ ----------
Efs_Mm      ---------- -PSLCPASPT QPG-SSCPTP ERGC------ ----------
Efs_Rn      ---------- -PSLCPAPPT QPGCSSCPTP ERGC------ ----------
p130Cas_Gg  -------QQQ QQQQQQQAAG PGQGQAPPVP QPTLP----- ----------
Consistency 0010112232 3533334455 3233555559 4534324221 2322100000

            .........260.........270.........280.........290.........300
NEDD9_Hs    ------SYQN QGIYQVPTGH GTQEQEVYQV PPSVQR---- -------SIG
NEDD9_Mm    ------SYQN QGIYQVPTGH GTPEQDVYQV PPSVQR---- -------NIG
NEDD9_Rn    ------SYQN QGIYQVPTGH GTPEQDVYQV PPSVQR---- -------NIG
NEDD9_Gg    ------SHTN QGIYQIPTGH GVAGQDIYQV PPSTQR---- -------CLD
p130Cas_Hs  TPHHPFPSPA TDLYQVPPGP GGPAQDIYQV PPSAGMGHDI YQVPPSMDTR
p130Cas_Mm  TPHHSFPSPA TDLYQVPPGP GSPAQDIYQV PPSAGIGHDI YQVPPSLDTR
p130Cas_Rn  TPHHSFPSPA TDLYQVPPGP GSPAQDIYQV PPSAGTGHDI YQVPPSLDTR
HEPL_Hs     ------SFPK QAILTLPRPV RASLPTLPSQ VYDVPT---- ----------
HEPL_Mm     ------SFPK QALSVLPRPT RASLPTLPSQ VYDVPV---- ----------
HEPL_Rn     ------NFPK QALFVLPRPT RASLPTLPSQ VYDVPV---- ----------
HEPL_Gg     ------CSTQ QHLFTLPRAC RASVPNITSE LYDVPSMQSR ESLLTQSGAT
Efs_Hs      --------ED QEVYVVPPPA RPCPTSGPPA GPCPPS---- ----------
Efs_Mm      --------EE QEVYVIPPPA RPCSASGLPA RSCSPS---- ----------
Efs_Rn      --------EE QEVYVIPPPA RPCSASGLPA RSCSPS---- ----------
p130Cas_Gg  --------YH HQGGYAPLSP ASQYTPMHPS YVPQGD---- ----------
Consistency 0000002244 747657*453 5443555455 3455440000 0000000111

            .........310.........320.........330.........340.........350
NEDD9_Hs    GTSGPHVGKK VITP-VRTGH GYVYEYPSRY QKDVYDIPP- --SHTTQGVY
NEDD9_Mm    GTNGPLLSKK VITP-VRTGH GYVYEYPSRY QKDVYDVPP- --SHSTQGVY
NEDD9_Rn    STNGPHLGKK VITP-VRVGH GYVYEHPSRY QKDVYDVPP- --SHTTQGVY
NEDD9_Gg    GPPLT---NK VITP-VRSGQ GYVYEFPSKY QKDTYDIPP- --VRPLQGIY
p130Cas_Hs  SWEGTKPPAK VVVP-TRVGQ GYVYEAAQPE Q-DEYDIPRH LLAPGPQDIY
p130Cas_Mm  GWEGTKPPAK VVVP-TRVGQ GYVYEAAQTE Q-DEYDTPRH LLAPGPQDIY
p130Cas_Rn  SWEGTKPPAK VVVP-TRVGQ GYVYEASQAE Q-DEYDTPRH LLAPGSQDIY
HEPL_Hs     ---QHRGPVV LKEP----EK QQLYDIPASP KKAG------ --LHPPDSQA
HEPL_Mm     ---QRQGFST LERL----EK QQFYDIPTSS QKAL------ --LHSSTSQA
HEPL_Rn     ---QRQVSST LERP----EK QQFYDIPTSS QKAV------ --F--HISTS
HEPL_Gg     PPAARKGSVL LRSPRCFQEE QQFYNVPSSS EKAGAVIQDS AVGNLYDVPS
Efs_Hs      ---------- ---------P DLIYKIPRAS ---------- ------GTQL
Efs_Mm      ---------- ---------S DSIYKVPRVN ---------- ------GMQL
Efs_Rn      ---------- ---------S DSIYKVPRVN ---------- ------GIQL
p130Cas_Gg  ---------- ---------- -NVYLMPVPG K--------- ----------
Consistency 1012221123 4224012134 447*547544 5232221210 0022124344

            .........360.........370.........380.........390.........400
NEDD9_Hs    DIPPSSAKGP VFSVPVGEIK PQGVYDIP-- --PTKGVYAI PPSACRDEA-
NEDD9_Mm    DIPPSSVKGP VFSVPVGEIK PQGVYDIP-- --PTQGVYAI PPSACRDEA-
NEDD9_Rn    DIPPSSVKGP VFSVPVGELK PQGVYDIP-- --PTQGVYAI PPSACRDEA-
NEDD9_Gg    DIPPTSAKGT AHSVPTGDAK ALGVYDVP-- --PAKGMYAT PPSTCRDDV-
p130Cas_Hs  DVPPVRGLLP SQYGQEVYDT PPMAVKGPNG RDPLLEVYDV PPSVEKGLPP
p130Cas_Mm  DVPPVRGLLP NQYGQEVYDT PPMAVKGPNG RDPLLDVYDV PPSVEKGLLS
p130Cas_Rn  DVPPVRGLLP NQYGQEVYDT PPMAVKGPNG RDPLLDVYDV PPSVEKGLPP
HEPL_Hs     SGQGVPLISV TTLRRGGYST -LPNPQ---- --KSEWIYDT PVSPGKASV-
HEPL_Mm     SGRDVTLAPT MAFRQGGGYN PLSSPQ---- --KSERIHDT PVLLEKADV-
HEPL_Rn     QGPGVTLAPT TAFRQGGGYN PLSSPQ---- --KSDWIHDT PVSLGKADV-
HEPL_Gg     KRETDASKKK SQKKYWGHCN TLPNPR---- --KSEWIYDI PISPEKTGL-
Efs_Hs      AAPR------ ---------- ---------- --DALEVYDV PPTALRV---
Efs_Mm      TASR------ ---------- ---------- --DVAEVYDV PPNILRA---
```

```
Efs_Rn      TAPR------ ---------- ---------- --DVGEVYDV PPNILRA---
p130Cas_Gg  ---------- ---------- ---------- --GQQGHYSG SAPS------
Consistency 4453222212 2221213212 3222220200 0045437866 9664273220

            .........410.........420.........430.........440.........450
NEDD9_Hs    GLR-EKYDFP PPMRQAGRPD L-RPEGV--- --YDIPPTCT KPAGKDLHVK
NEDD9_Mm    GLREKEYDFP PPMKQDGKPD T-RPEGV--- --YDIPPTST KTAGKDLHIK
NEDD9_Rn    GLREKEYDFP PPMKQDGKLD T-RPEGV--- --YDIPPTST KTVGKDLHTK
NEDD9_Gg    GLRDNLQDFS SPAGHN---- A-RQEGV--- --YDIPPPIT KATGKELIKK
p130Cas_Hs  SNHHAVYDVP PSVSKDVPDG PLLREET--- --YDVPPAFA KAKPFDPART
p130Cas_Mm  SSHHSVYDVP PSVSKDVPDG PLLREET--- --YDVPPAFA KPKPFDPTRH
p130Cas_Rn  SNHHSVYDVP PSVSKDVPDG PLLREET--- --YDVPPAFA KPKPFDPTRH
HEPL_Hs     RNTPLTSFAE ESRPHALPSS SSTFYNPPSG RSRSLTPQLN NNVPMQKKLS
HEPL_Mm     RNVSMTSFTK DSGSRAIPGS SAVHTGA--- --VALSPQLG NTVQRKNSLP
HEPL_Rn     RNASMTSFTK ESCSNTIPGS SVVRIGA--- --VTLSPQLG NAVQKKSSPP
HEPL_Gg     KQNSSVHSME NQVLYDIPP- ARYKALTTNA EANVVNPQLY DIPPTQRKLT
Efs_Hs      ---------- ---------- --PSSGP--- --YDCPASFS HPLTRVAPQP
Efs_Mm      ---------- ---------- --PSSCP--- --YDSPASFS CPVAPVVPQP
Efs_Rn      ---------- ---------- --PSNCP--- --YDSPASFS CPATPVVPQP
p130Cas_Gg  ---------- ---------- ---------- --GQFPPAPA KQPPAY----
Consistency 2221222222 2322222211 2033434000 0056567545 4444343333

            .........460.........470.........480.........490.........500
NEDD9_Hs    YNCDIPGAAE --PVA-RRHQ SLSPNHPPPQ LGQSV---GS QNDAYDVPRG
NEDD9_Mm    FPCDAPGGVE --PMA-RRHQ SFSLHHAPSQ LGQSG---DT QSDAYDVPRG
NEDD9_Rn    FPCDAPGGAE --PMA-RRHQ SLSLHHAPSQ VGPSG---DT QSDAYDVPRG
NEDD9_Gg    FSSEGLALTD GAPHK-QSVY DIPVNHQNHF LGQQI---AP QKDVYDTPRG
p130Cas_Hs  PLVLAAPPPD S-PPA-EDVY DVPPPAPDLY DVPPGLRRPG PGTLYDVPRE
p130Cas_Mm  PLILAAPPPD S-PAA-EDVY DVPPPAPDLY DVPPGLRRPG PGTLYDVPRE
p130Cas_Rn  PLILAAPPPD S-PPA-EDVY DVPPPAPDLY DVPPGLRRPG PGTLYDVPRE
HEPL_Hs     LPEIPSYGFL VPRGTFPLDE DVSYKVPSSF LIPRVEQQNT KPNIYDIPKA
HEPL_Mm     EEPTYAFPTS RDP-L-PSDA GGSYKVPSRF LIPRVEQQNT MPNIYDTPKA
HEPL_Rn     EETSYAFLTP RGDSL-PSDA GSSYKVPSRF LIPRVERQNT KPNIYDTPKA
HEPL_Gg     FPDVPLYDVP SSKDV-LLQQ NGSCDVPPSL PAPNAENQIS GGNVYDIPKG
Efs_Hs      PGEDD----- ---------- ---------- ---------- --APYDVPLT
Efs_Mm      PREDE----- ---------- ---------- ---------- --APYDVPLA
Efs_Rn      PGEDE----- ---------- ---------- ---------- --APYDVPLA
p130Cas_Gg  ---------- ---------- ---------- ---------- ---PKQTPP-
Consistency 3223321122 1031302212 2231223222 2232201112 2244897*54

            .........510.........520.........530.........540.........550
NEDD9_Hs    VQF------L EPPAETSEKA NPQERDGVYD VPLHNPPDAK GSRDLVDGIN
NEDD9_Mm    VQF------L EVPTETSEKA NPEERDGVYD VPLHNPADAK GSRDVVDGIN
NEDD9_Rn    VQF------L EVPTESSEKA NLEERDGVYD VPLHNPADAK GSRDVVDGIN
NEDD9_Gg    IAF------P GQQTGLNESL ASEGREGVYD VPPPVLQDTK GLQDVTDGMN
p130Cas_Hs  ---------R VLPPEVADGG VVD--SGVYA VPPP------ AEREAPAEGK
p130Cas_Mm  ---------R VLPPEVADGS VVD--DGVYA VPPP------ AEREAPTDGK
p130Cas_Rn  ---------R VLPPEVADGS VID--DGVYA VPPP------ AEREAPTDGK
HEPL_Hs     TSS-----VS QAGKELEKAK EVSEN-SAGH NSSWFSR--- RTTSPSPEPD
HEPL_Mm     MQGVSHNAPK AMQGVSLAGK ELERGREAPE NSPWISG--- QTSFLSPDSD
HEPL_Rn     MQGVFQNTSK ATQGVSLAGK ELERGREAPE NSPWVSR--- QTNFLSPDSD
HEPL_Gg     LPT------A MQPKKEMEKN SNHSGNQAYH IPTQLSR--- DTKPEHDRLP
Efs_Hs      PKP-----PA ELEPDLEWEG GREPGPPIYA APS------- ----------
Efs_Mm      LKP-----PA ELERDPEWEG GREPGPPLYA APS------- ----------
Efs_Rn      LKS-----PA EMEPDLEWEG GREPGPPLYA APS------- ----------
p130Cas_Gg  ---------H AFPSAGQE-- -------IYQ VP-------- ----------
Consistency 3310000004 4454544443 3352234664 5731111000 2232222212

            .........560.........570.........580.........590.........600
NEDD9_Hs    RLSFS-STGS TRSNMSTSST SSKESSLSAS PAQD-KRLFL DPDTAIERLQ
NEDD9_Mm    RLSFS-STGS TRSNMSTSST SSKESSLSAS PSQD-KRLRL DPDTAIEKLY
NEDD9_Rn    RLSFS-STGS TRSNMSTSST SSKESSLSAS PSQD-KRLLL DPDTAIGKLY
NEDD9_Gg    RLSFS-STGS TRSNMSTSST TSKDSSFSAS TTQD-KRLIL DPDTAIERLY
p130Cas_Hs  RLSAS-STGS TRSSQSASSL EVAGP----- -----GREPL ELEVAVEALA
p130Cas_Mm  RLSAS-STGS TRSSQSASSL EVVVP----- -----GREPL ELEVAVESLA
p130Cas_Rn  RLSAS-STGS TRSSQSASSL EVVVP----- -----GREPL ELEVAVETLA
HEPL_Hs     RLSGS-SSDS RASIVSSCST TSTDDSSSSS SEESAKELSL DLDVAKETVM
HEPL_Mm     RLSVA-SSDS RASVVSSCSS ISMDSSSGSS SEDSVKELWM DVDFAKETAV
HEPL_Rn     RLSVA-SSDS RASVVSSCSS LSTDSSPGSS SEDSVKELWM DLDFAKETAV
HEPL_Gg     MCGHDNSADS KNSTLSMSSN SSSESSSASS SDEPDKEIKL DLEVAIETLT
Efs_Hs      ---------- ---------- ---------- --------NL KRASALLNLY
Efs_Mm      ---------- ---------- ---------- --------NL KRASALLNLY
Efs_Rn      ---------- ---------- ---------- --------NL KRASALLNLY
p130Cas_Gg  ---------- ---------- ---------- ---------- ---PSLSQAA
Consistency 4442405335 3352353352 2322321122 1111033327 5354965574
```

```
            .........610 .........620 .........630 .........640.........650
NEDD9_Hs    RLQQALEMGV SSLMALVTTD -----WRCY- GYMERHINEI RTAVDKVELF
NEDD9_Mm    RLQQTLEMGV CSLMSLVTTD -----WRCY- GYMERHINEI RTAVDKVELF
NEDD9_Rn    WLQQTLETGI CSLMSLVTID -----WRSY- GYMERHINEI RTAVDKVELF
NEDD9_Gg    RLQQMVETAV NNLMAFATAD -----WRSY- SYMEKHINEI HTAVDKVEQS
p130Cas_Hs  RLQQGVSATV AHLLDLAGSA GATGSWRSPS EPQEPLVQDL QAAVAAVQSA
p130Cas_Mm  RLQQGVSTTV AHLLDLVGSA SGPGGWRGTS EPQEPPAQDL KAAVAAVHGA
p130Cas_Rn  RLQQGVSTTV AHLLDLVGSA SGPGGWRSTS EPQEPPVQDL KAAVAAVHGA
HEPL_Hs     ALQHKVVSSV AGLMLFVSRK -----WRFR- DYLEANIDAI HRSTDHIEES
HEPL_Mm     SLQHKVASSA AGLLLFVSRT -----WRFK- DSLETNIHRI RRAADHVEES
HEPL_Rn     ALQSRVASSV AGLLLFVSRT -----WRFK- DSLETNVNRI RGAADRVEES
HEPL_Gg     RLQHSVSSSV ASLMIFVSSK -----WRLQ- EHLEKNIEEI HRAIDHIKVS
Efs_Hs      EAPEELLADG EG-------- ---------- GGTDEGIYDV PLLGPEAPPS
Efs_Mm      EAPEELLANG ES-------- ---------- RDADEGIYDV PLLGPEPPSP
Efs_Rn      EAPEELLANG ES-------- ---------- RDADEGIYDV PLLGPEPPSP
p130Cas_Gg  DAYPVGSAS- ---------- ---------- --PPQDIYQV PPSVAQAQDI
Consistency 4765374655 4454234322 0000055210 4347438468 4475546534

            .........660 .........670 .........680 .........690.........700
NEDD9_Hs    LKEYLHFVKG AVANAACLPE LILHNKMKRE LQRVEDSHQI LSQTSHDLNE
NEDD9_Mm    LREYLHFAKG ALANASCLPE LVLHNKMKRE LQRVEDSHQI LSQTSHDLNE
NEDD9_Rn    LREYLHFARG ALANASCLPE PVLLNKMNRE LQRVDDSHQI LSQTSHDLNE
NEDD9_Gg    LLEYLQFAKG SAANASFLSE ISLHNKMRRE VQRLEDSHQI LTQTSHDLNS
p130Cas_Hs  VHELLEFARS AVGNAAHTSD RALHAKLSRQ LQKMEDVHQT LVAHGQALDA
p130Cas_Mm  VHELLEFARG AVSNATHTSD RTLHAKLSRQ LQKMEDVYQT LVVHGQVLDS
p130Cas_Rn  VHELLEFARS AVSSATHTSD RTLHAKLSRQ LQKMEDVYQT LVVHGQVLDS
HEPL_Hs     VREFLDFARG VHGTACNLTD SNLQNRIRDQ MQTISNSYRI LLETKESLDN
HEPL_Mm     VREFLDFAQG VGGTACNLTD SYLQARIRDQ LQTISSSYQT LLDAKGSLDR
HEPL_Rn     LREFLDFAQG VGGLACNLTD SYLQARVRDQ LQTISSSYQT LLDAKGTLDR
HEPL_Gg     LGEFLAFARA LKVNASYLTD NNLQIRIKKQ LEILMNSFQI LTETREALNN
Efs_Hs      PEPPGALASH DQDTLAQLLA RSPPPPHRPR LPSAESLSRR PLPALPVPEA
Efs_Mm      EPPVASSST- DLDTVAQLPT RSSPPQHRPR LPSTESLSRR PLPALPVSEA
Efs_Rn      EPPAASSSA- DPDTVAQLLT RSPPPQHRPR LPSTESLSRR PLPALPVSEA
p130Cas_Gg  YQVPPSLDAR SWEGP----- KPSGKVLLPT RVGQVYVYDS PKGEQDEYDF
Consistency 5464646754 4345753645 4464465546 8645655574 6545444664

            .........710 .........720 .........730 .........740.........750
NEDD9_Hs    CSWSLNILAI NKPQNKCDDL DRFVMVAKTV PDDAKQLTTT INTNAEALFR
NEDD9_Mm    CSWSLNILAI NKPQNKCDDL DRFVMVAKTV PDDAKQLTTT ISTYAETLFR
NEDD9_Rn    CSWSLNVLAI NKPQNKCDDL DRFVMVAKTV PDDAKQLTTT ISTYAETLFR
NEDD9_Gg    YNWSLNVLAV NRLQNKCDDL DRFVMVAKTV PDDAKQLTTT ISVNAELLFK
p130Cas_Hs  GRGGSGAT-- ------LEDL DRLVACSRAV PEDAKQLASF LHGNASLLFR
p130Cas_Mm  GRGSPGFT-- ------PEDL DRLVACSRAV PEDAKQLASF LHGNASLLFR
p130Cas_Rn  GRGGPGFT-- ------LDDL DRLVACSRAV PEDAKQLASF LHGNASLLFR
HEPL_Hs     RNWPLEVLVT DSVQNSPDDL ERFVMVARML PEDIKRFASI VIANGRLLFK
HEPL_Mm     CNWSLEVLVT DKVQNSLDDL ERFVATARIV PEDVKRFTSI VIANGKLLFK
HEPL_Rn     CNWSLEVLVV DKAQSSVDDL ERFVQVARVV PEDVKRFTSI VVANGKLLFK
HEPL_Gg     CNWSLEALVL KKPYSNPDDL DRFVMVARTI PDDLKRFVSI IIANGKLLFR
Efs_Hs      PSPSPVPS-- ------PAPG RKGSIQDRPL PPPPPRLPGY GGPKVEGDPE
Efs_Mm      PAPSPAPS-- ------PAPG RKGSIQDRPL PPPPPCLPGY GGLKPEGDPE
Efs_Rn      PAPSPAPS-- ------PAPG RKGSIQDRPL PPPPPCLPGY GGLKPEGDPE
p130Cas_Gg  ---------- ---------- ---------- SRHLLSLGS- ----------
Consistency 2436434511 1212213565 5746445747 9565657564 4335454556

            .........760 .........770 .........780 .........790.........800
NEDD9_Hs    PGPGSLHLKN GPESIMNSTE YPHGGSQGQL LHPGDHKAQA HNKALP--PG
NEDD9_Mm    ADPANSHLKN GPNSIMNSSE YTHPGSQMQP LHPGDYKAQV HSKPLP--PS
NEDD9_Rn    VDPGNSHLKN GPDSITNSIE YTHTGSQMQP LRPGDYKGQV HNKPLP--PT
NEDD9_Gg    QVSGSSRFRS VPENMNA--- -PDYAYNSPH MQRHGEKPQN HFSSLP--PL
p130Cas_Hs  RTKATAPGP- ---------- ---EGGGTLH PNPTDKTSSI QSRPLPSPPK
p130Cas_Mm  RTKAPGPGP- ---------- ---EGSSSLH PNPTDKASSI QSRPLPSPPK
p130Cas_Rn  RTKAPGPGP- ---------- ---EGSSSLH LNPTDKASSI QSRPLPSPPK
HEPL_Hs     RNCEKEE--- ---------- -----TVQLT PNAEFKCEKY IQPP------
HEPL_Mm     QNCEKGE--- ---------- -----M-DLK CERCIRPPQR ET--------
HEPL_Rn     QNCERGE--- ---------- -----V-DWR CERCIQPPQR EAESH-----
HEPL_Gg     KNEKEQE--- ---------- -----MKQPR VSPGYKMPKQ ITSP------
Efs_Hs      GR-------- ---------- -------EME DDPAGHHNEY EGIP------
Efs_Mm      CR-------- ---------- -------EVA NDPAGPHNEY EGIP------
Efs_Rn      CR-------- ---------- -------EVD SDSGGPHNEY EGIP------
p130Cas_Gg  ---------- ---------- ---------- ---------- ----------
Consistency 3312222010 0000000000 0000121433 2453333353 4435220020

            .........810 .........820 .........830 .........840.........850
NEDD9_Hs    LSKEQAPDCS SSDGSERSWM DDYDYVHLQG KEEFERQQKE LLEKENIMKQ
```

```
NEDD9_Mm     LSKDQPPDCG SSDGSERSWM DDYDYVHLQG KEEFERQQKE LLEKENIMKQ
NEDD9_Rn     LSKEQPPDCS SSDGSERSWM DDYDYVHLQG KEEFERQQKE LLEKENIMKQ
NEDD9_Gg     LSKGQQPHST ASESSEKSWM DDYDYVHLQG KEEFERQQKE LLEKENIIKQ
p130Cas_Hs   FTSQDSPD-G QYENSEGGWM EDYDYVHLQG KEEFEKTQKE LLEKGSITRQ
p130Cas_Mm   FTSQDSPD-G QYENSEGGWM EDYDYVHLQG KEEFEKTQKE LLERGNIMRQ
p130Cas_Rn   FTSQDSPD-G QYENSEGGWM EDYDYVHLQG KEEFEKTQKE LLEKGNIVRQ
HEPL_Hs      ---------- ------QRET ESHQKSTPST KQREDE---- ----------
HEPL_Mm      ---------- ---------- ESYQESSPFD RQPTTE---- ----------
HEPL_Rn      -------PER SSPFHKQPAA EHSLELAKKS RGDVCGQKSP NLQ-------
HEPL_Gg      ---------- -----RRIEI DSLPVSAPDK PNPSQGS--- ----------
Efs_Hs       ---------- --------MA EEYDYVHLKG MDKAQGSRPP D---------
Efs_Mm       ---------- --------MA EEYDYVHLKG VDTAQGSRPL D---------
Efs_Rn       ---------- --------MA EEYDYVHLKG VDKAQGSRPL D---------
p130Cas_Gg   ---------- ---------Q EIXDVPPVRG ALPSQVSQGT KFNH-----F
Consistency  1111112101 1111222134 85-2147483 6486565556 5554644433 2322112112
```

```
             .........860 .........870 .........880 .........890.........900
NEDD9_Hs     NKMQLEHHQL SQFQLLEQEI TKPVENDISK WKPSQSL-PT TNSG-VSAQD
NEDD9_Mm     SKAQLEHHQL SQFQLLEQEI TKPVENDISK WKPSQSL-PT TNNS-VGAQD
NEDD9_Rn     SKAQLEHHQL SQFQLLEQEI TKPVENDISK WKPSQSL-PT TNNS-VGAQD
NEDD9_Gg     SKTELEHHQI NQFQRLEQEI TKPVENDISK WKPPQAL-QT ANST-IAFHD
p130Cas_Hs   GKSQLELQQL KQFERLEQEV SRPIDHDLAN WTPAQPLAPG RTGG-LGPSD
p130Cas_Mm   GKGQLELQQL KQFERLEQEV SRPIDHDLAN WTPAQPLVPG RTGG-LGPSD
p130Cas_Rn   GKGQLELQQL KQFERLEQEV SRPIDHDLAN WTPAQPLVPG RTGG-LGPSD
HEPL_Hs      ---------- HSSELLKKNR -----ANICG QNPG----PL IPQP-SSQQT
HEPL_Mm      ---------- HSFELARKNR -----VNVCW QSPP----PL TSPS-PSGQN
HEPL_Rn      ------KKGK NTMEGKSNEN -----QDFCG TSPT----PL PSPS-PSGQN
HEPL_Gg      -------NET TKENVSEDCE -----YVQLQ VLPS----TK KNAV-RSQQD
Efs_Hs       ------QACT GDPELPERGM ---------- PAPQEALSPG EPLV-VSTGD
Efs_Mm       ------KAFP VDPELLERGL ---------- AERKEALSPE EPLV-LSTGD
Efs_Rn       ------NAFP ADPELLERGL ---------- VEQKEALSPE EPLV-LSTGD
p130Cas_Gg   YVTPIYSTQK KQPERREQEE KRPKDTDGSK GSPPQQYGPE RGGGALCAAD
Consistency  1212222333 4647558654 1221224332 3484424083 4434056357
```

```
             .........910 .........920 .........930 .........940.........950
NEDD9_Hs     RQLLCFYYDQ CETHFISLLN AIDALFSCVS SAQPPRIFVA HSKFVILSAH
NEDD9_Mm     RQLLCFYYDQ CETHFISLLN AIDALFSCVS SAQPPRIFVA HSKFVILSAH
NEDD9_Rn     RQLLCFYYDQ CETHFISLLN AIDALFSCVS SAQPPRIFVA HSKFVILSAH
NEDD9_Gg     RQPLLFYSDQ YETHFNSLLN TIDAFFSCVS ASQPPRIFVA HSKLVILSAH
p130Cas_Hs   RQLLLFYLEQ CEANLTTLTN AVDAFFTAVA TNQPPKIFVA HSKFVILSAH
p130Cas_Mm   RQLLLFYLEQ CEANLTTLTD AVDAFFTAVA TNQPPKIFVA HSKFVILSAH
p130Cas_Rn   RQLLLFYLEQ CEANLTTLTD AVDAFFTAVA TNQPPKIFVA HSKFVILSAH
HEPL_Hs      PERKPRLSEH CRLYFGALFK AISAFHGSLS SSQPAE-IIT QSKLVIMVGQ
HEPL_Mm      TERKIHLSKH SRLYFGALFK AISVFASSLS NGQPPEVFIT QSKLVITVGQ
HEPL_Rn      VERKIRLSEH SRLYFGALFK AIGVFASSLS NSQPPEVFIT QSKLAITIGQ
HEPL_Gg      SAKKVVIPEQ CRLCFGALHK AIAVFNSSLS SKQPPEVFIS HSKLIIMVGQ
Efs_Hs       LQLLYFYAGQ CQSHYSALQA AVAALMSSTQ ANQPPRLFVP HSKRVVVAAH
Efs_Mm       LQLLHFYAGQ CQSHYSALQA AVAALVASTQ ANQPPCLFVP HGKRVVVAAH
Efs_Rn       LQLLHFYAGQ CQSHYSALQT AVAALMSSTQ ANQPPCLFVP HGKRVVVAAH
p130Cas_Gg   RQLLLFYLEQ CEANLTTLTN AIDAFFTAIS TNQPPKIFVA HSKFIILSAH
Consistency  4856467457 775564 6*45 9958747667 65**957996 78*5996677
```

```
             .........960 .........970 .........980 .........990.........1000
NEDD9_Hs     KLVFIGDTLT RQVTAQDIRN KVMNSSNQLC EQLKTIVMAT KMAALHYPST
NEDD9_Mm     KLVFIGDTLT RQVAAQDIRN KVRNSSNQLC EQLKTIVMAT KMAALHYPST
NEDD9_Rn     KLVFIGDTLT RQVAAQDIRN KVRNSSNQLC EQLKTIVMAT KMAALHYPST
NEDD9_Gg     KLVFIGDTLT RQLTSQDIRN RVRNSSDQLC ELLKSIVMAT KMAALHYPHT
p130Cas_Hs   KLVFIGDTLS RQAKAADVRS QVTHYSNLLC DLLRGIVATT KAAALQYPSP
p130Cas_Mm   KLVFIGDTLS RQAKAADVRS QVTHYSNLLC DLLRGIVATT KAAALQYPSP
p130Cas_Rn   KLVFIGDTLS RQAKAADVRS KVTHYSNLLC DLLRGIVATT KAAALQYPSP
HEPL_Hs      KLV---DTLC METQERDVRN EILRGSSHLC SLLKDVALAT KNAVLTYPSP
HEPL_Mm      KLV---DTLC SETQEKDERN EILCGSSHLC GLLKDLALAT KSAVIQYPSP
HEPL_Rn      KLV---DTLC SETQERDERN EILCGSSHLC GLLKDLALAT KNAVIQYPSP
HEPL_Gg      KIV---DSLC QDTQLREARS DVLHSSSRFC SLLKNLALAT KNAAIQYPNA
Efs_Hs       RLVFVGDTLG RLAASAPLRA QVRAAGTALG QALRATVLAV KGAALGYPSS
Efs_Mm       RLVFVGDTLG RLAASAALRA QVGAAGTMLA QTLRATVLAV KGAALGYPSD
Efs_Rn       RLVFVGDTLG RLAASAPLRA QVGAAGTVLA QTLRATVLAV KGAALGYPSD
p130Cas_Gg   KLVFIGDTLS RQAKAQDVRH KVTHYSNLLC ELLKEIVVST KAAALHYPSP
Consistency  89*545*9*4 75656566*5 6944486497 55*8477678 *4*894**85
```

```
             .........1010.........1020.........1030
NEDD9_Hs     TALQEMVHQV TDLSRNAQLF KRSLLEMATF
NEDD9_Mm     TALQEMVHQV TDLSRNAQLF KRSLLEMATF
NEDD9_Rn     TALQEMVHQV TDLSRNAQLF KRSLLEMATF
NEDD9_Gg     AALQEMVNRV TELSYHAQLF KLSLVQMASL
```

Results colour-coded for amino acid conservation

```
p130Cas_Hs  SAAQDMVERV  KELGHSTQQF  RRVLGQLAAA
p130Cas_Mm  SAAQDMVDRV  KELGHSTQQF  RRVLGQLAAA
p130Cas_Rn  SAAQDMVDRV  KELGHSTQQF  RRVLGQLAAA
HEPL_Hs     AALGHLQAEA  EKLEQHTRQF  RGTLG-----
HEPL_Mm     SALSLLQSEV  ERLEHHSRKF  RDTLE-----
HEPL_Rn     SALGLLQSEL  EKLEYHSRKF  RGTLE-----
HEPL_Gg     DAMRELQNKN  DELYKYTQQF  RAMLE-----
Efs_Hs      PAIQEMVQCV  TELAGQALQF  TTLLTSLAP-
Efs_Mm      TAVQEMARCV  AELAGQALRF  TTLLAGLLP-
Efs_Rn      TAVQEMARCV  AELAGQALRF  TTLLAGLLP-
p130Cas_Gg  AASKDMVERV  KDLANSTQQF  RMVLGQLAAM
Consistency 6*66586447  56*545665*  645*334421
```
